# Evaluation of OpenArray™ as a Genotyping Method for Forensic DNA Phenotyping and Human Identification

**DOI:** 10.3390/genes12020221

**Published:** 2021-02-03

**Authors:** Michele Ragazzo, Giulio Puleri, Valeria Errichiello, Laura Manzo, Laura Luzzi, Saverio Potenza, Claudia Strafella, Cristina Peconi, Fabio Nicastro, Valerio Caputo, Emiliano Giardina

**Affiliations:** 1 Department of Biomedicine and Prevention, Tor Vergata University of Rome, 00133 Rome, Italy; michele.ragazzo@uniroma2.it (M.R.); giuliopuleri@gmail.com (G.P.); valeria.errichiello@uniroma2.it (V.E.); laura.manzo@uniroma2.it (L.M.); lauraluzzi92@gmail.com (L.L.); claudia.strafella@gmail.com (C.S.); v.caputo91@gmail.com (V.C.); 2 Department of Biomedicine and Prevention, Section of Legal Medicine, Social Security and Forensic Toxicology, University of Rome Tor Vergata, 00133 Rome, Italy; potenza@med.uniroma2.it; 3 Genomic Medicine Laboratory UILDM, IRCCS Santa Lucia Foundation, 00179 Rome, Italy; cristinapeconi@gmail.com; 4 Austech, 00153 Rome, Italy; f.nicastro@austech-italy.com

**Keywords:** TaqMan SNP genotyping, OpenArray™ system, forensic DNA phenotyping, forensic DNA typing

## Abstract

A custom plate of OpenArray™ technology was evaluated to test 60 single-nucleotide polymorphisms (SNPs) validated for the prediction of eye color, hair color, and skin pigmentation, and for personal identification. The SNPs were selected from already validated subsets (Hirisplex-s, Precision ID Identity SNP Panel, and ForenSeq DNA Signature Prep Kit). The concordance rate and call rate for every SNP were calculated by analyzing 314 sequenced DNA samples. The sensitivity of the assay was assessed by preparing a dilution series of 10.0, 5.0, 1.0, and 0.5 ng. The OpenArray™ platform obtained an average call rate of 96.9% and a concordance rate near 99.8%. Sensitivity testing performed on serial dilutions demonstrated that a sample with 0.5 ng of total input DNA can be correctly typed. The profiles of the 19 SNPs selected for human identification reached a random match probability (RMP) of, on average, 10^−8^. An analysis of 21 examples of biological evidence from 8 individuals, that generated single short tandem repeat profiles during the routine workflow, demonstrated the applicability of this technology in real cases. Seventeen samples were correctly typed, revealing a call rate higher than 90%. Accordingly, the phenotype prediction revealed the same accuracy described in the corresponding validation data. Despite the reduced discrimination power of this system compared to STR based kits, the OpenArray™ System can be used to exclude suspects and prioritize samples for downstream analyses, providing well-established information about the prediction of eye color, hair color, and skin pigmentation. More studies will be needed for further validation of this technology and to consider the opportunity to implement this custom array with more SNPs to obtain a lower RMP and to include markers for studies of ancestry and lineage.

## 1. Introduction

Short tandem repeats (STRs) are genetic variants widely used in forensic investigations for the identification of offenders and in cases concerning relationship testing [1,2]. However, the multiplexing capacity of STRs is relatively low because of the need for capillary electrophoresis and inability to accommodate large numbers of markers. In the genomic era, it is well known that STRs are not the only source of genetic variation in humans [3]. Single-nucleotide polymorphisms (SNPs) are the most common type of genetic variation among people and can provide additional information such as the inferred ancestry of a DNA sample and phenotypes (eye and hair color) [4]. From a technical point of view, SNPs can be used to support forensic DNA analyses because of an abundance of potential markers, amenability to automation, and potential reduction in required fragment length to only 60–80 bp [5,6,7].

Forensic analysis usually involves comparisons between genetic profiles extracted from biological samples collected from a crime scene, and objects and suspects which are thought to be associated to that crime. In the absence of suspects, the power of forensic DNA analysis has been enhanced through the development of DNA databases, allowing the identification of unknown offenders and serial offenders by linking different crimes [1,8,9]. However, in criminal cases without an STR profile match, police investigation can be aided by different kinds of genetic tests.

In this context, the analysis of SNPs for the study of phenotypic variability (forensic DNA phenotyping, FDP) and for the determination of biogeographical origin is particularly promising [1,10]. The main advantage of such analysis is to narrow down the number of potential crime scene trace donors to a smaller group of people who most likely have the externally visible characteristics and biogeographic ancestry that were inferred from the crime scene DNA [11].

Forensic DNA phenotyping is explicitly regulated and permitted by law in two EU member states (the Netherlands and Slovakia) and practiced in compliance with existing laws in six more (Poland, Czech Republic, Sweden, Hungary, Austria, and Spain) and the United Kingdom [11].

In the “omic era”, different approaches have been developed to test thousands of genetic variations for forensic purposes (phenotype, ancestry, and identification). Real-time PCR using TaqMan chemistry is a well-known technology routinely used in the workflow of forensic laboratories to quantify the DNA extracted from biological samples [12,13]. The OpenArray™ system (Thermo Fisher Scientific, Waltham, MA, USA) applies TaqMan chemistry for the simultaneous and massive study of samples and SNPs [14,15].

Here we report a pilot study aimed at evaluating the applicability of OpenArray™ technology (Thermo Fisher Scientific, Waltham, MA, USA) for the testing of SNPs for human identification and for inferring phenotypes. To this end, we selected the entire panel of 41 SNPs for eye, hair, and skin color prediction from the validated tool HIrisPlex-S and 19 SNPs for individual identification from the Precision ID Identity SNP Panel (Thermo Fisher Scientific, Waltham, MA, USA) and ForenSeq DNA Signature Prep Kit (Verogen Inc, San Diego, CA, USA). A total of 314 samples were typed to test the genotyping accuracy of OpenArray™ through comparison with resequencing data.

The sensitivity of the platform was assessed using four serial dilutions of DNA consisting of 10, 5, 1, and 0.5 ng [16,17,18].

## 2. Materials and Methods

### 2.1. Selection of SNPs

In this work we selected 41 SNPs for eye, hair, and skin color prediction from the HIrisPlex-S system and 19 SNPs from a universal individual identification SNPs panel validated for forensic purposes [16,17,18,19]. The SNPs for human identification were selected based on their chromosomal location (each SNP is mapped to a different chromosome to avoid linkage and linkage disequilibrium effects). These genetic variants were included in a customized panel of 60 SNPs able to provide both human identification and phenotyping information in the same analytical session. The TaqMan probe related to rs1805008 could not be developed. Therefore, rs11538871 was chosen because of its complete linkage disequilibrium (LD) with rs1805008 [20].

The selected SNPs are summarized in Table 1.

### 2.2. Ethical Committee Approval

These investigations were carried out following the rules of the Declaration of Helsinki of 1975, revised in 2013. According to Point 23 of this declaration, approval from the ethical committee of Policlinico Tor Vergata (protocol number: Prot. CE/PROG.177/20) was obtained.

### 2.3. Reference DNA Samples

A total of 314 reference DNA samples were used. In particular, 114 genomic DNA samples were obtained from the 1000 Genomes Project (Coriell Institute for Medical Research, Camden, NJ, USA) and 200 blood samples were obtained from the Laboratory of Genomic Medicine UILDM (Italian Muscular Dystrophy Association) Santa Lucia Foundation [21]. The 114 samples from 1000 Genomes were selected from a small town near Florence in the Tuscany region of Italy. The genotype data of genomic DNA samples from 1000 Genomes were extracted from the Ensembl genome browser [22]. An additional four volunteer donors were used for the sensitivity testing.

### 2.4. Biological Samples from Real Cases

We analyzed a total of 21 samples of biological evidence that had single STR profiles generated during the routine workflow and that were assigned to their reference perpetrator. This evidence was from different tissues (10 from saliva, 2 from semen, 7 from blood, 2 from urine) and was attributed to 8 different subjects from different tissues [23,24]. All samples were marked with a specific internal code, as shown in Table 2 [2]. The forensic samples were loaded in the same plate twice with the purpose of verifying the reproducibility of genotyping assignation.

### 2.5. DNA Purification and Quantification

In accordance with the quality standards of forensic laboratories that require the use of automatized instruments, the genomic DNA of 225 samples were extracted using the Maxwell^®^16 MDx Instrument (Promega, Madison, WI, USA) and the DNA IQ Casework Pro Kit for Maxwell^®^16 (Promega, Madison, WI, USA).

Each DNA sample was quantified using the Quantifiler^®^Trio DNA Quantification Kit (Thermo Fisher Scientific, Waltham, MA, USA).

### 2.6. OpenArray™ Technology

OpenArray™ Technology (Thermo Fisher Scientific, Waltham, MA, USA) is a high-throughput real-time PCR genotyping method that allows for rapid screening of several TaqMan assays in many samples [13]. This real-time method involves the use of an array composed of 3072 through-holes running on the QuantStudio 12K Flex Real Time PCR System (Thermo Fisher Scientific, Waltham, MA, USA) with an OpenArray™ block. The OpenArray™ system is composed of a specific plate (OpenArray™ plate) enabling 192 samples to be typed in a single run [14].

For each sample, 5 ng (volume of 3 μL) of extracted DNA from the reference samples and 3 μL of 2× TaqMan OpenArray™ Genotyping Master Mix were manually loaded into 384 well-plates according to the manufacturer’s instructions (Thermo Fisher Scientific, Waltham, MA, USA). A negative control was obtained by adding 3 μL of pure distilled water to the Master Mix. The QuantStudio 12K Flex OpenArray™ AccuFill System transferred the previously generated mix to the TaqMan OpenArray™ plate. The amplification was performed using the QuantStudio 12K Flex Real Time PCR System (Thermo Fisher Scientific, Waltham, MA, USA) instrument, and the results were analyzed using TaqMan Genotyper Software (Thermo Fisher Scientific, Waltham, MA, USA) [14]. For each SNP, the call rate and concordance rate were calculated as described in Section 2.7 [25].

### 2.7. Sensitivity

For sensitivity studies, genotyping results from four volunteer donors were assessed from serial dilutions of DNA consisting of 10 ng, 5 ng, 1 ng, and 0.5 ng. These four volunteer donors provided a total of one sample (buccal swab) each.

### 2.8. Resequencing Analysis

The genotyping accuracy was assessed through comparison with resequencing data. Resequencing data were available from the Ensembl genome browser for the 1000 Genomes Project samples and from an internal database for the Laboratory of Genomic Medicine UILDM Santa Lucia Foundation samples. The PCR sequence reactions were performed with BigDye Terminator v3.1 (Thermo Fisher Scientific, Waltham, MA, USA) and the purification with BigDyeXTerminator (Thermo Fisher Scientific, Waltham, MA, USA) according to the manufacturer’s instructions. The samples were run on ABI3130xl (Thermo Fisher Scientific, Waltham, MA, USA) and the obtained sequences were read by Sequencing Analysis software v5.2 (Thermo Fisher Scientific, Waltham, MA, USA) [13].

### 2.9. Statistical Analysis

At the end of the analytical assay, TaqMan Genotyper Software indicated the percentage of successful genotyping, reported as the call rate. The call rate is defined as the percentage of SNPs that were assigned a genotype call by the software out of the total number of SNPs typed. According to the software’s instructions, a successful call rate is more than 90%. The concordance rate is defined as the proportion of SNPs typed by OpenArray™ confirming the resequencing data. This value was determined by comparing the allele calls generated by OpenArray™ technology with the genotypes resulting from resequencing data [25]. Concordance is the percentage of SNPs with identical results using both OpenArray™ and resequencing.

For phenotypic characterization of biological forensic samples (*n* = 17), we constructed a statistical prediction model using HIrisPlex-S webtool systems, available at https://hirisplex.erasmusmc.nl/ (accessed on 20 January 2021). Each phenotype was predicted by applying established algorithms for the color of the eyes, hair, and skin, which returns a *p*-value (prediction probability) [19]. In terms of eye color, the highest *p*-value is assigned to the most probable phenotype [19,26]. For the interpretation of hair color, we considered the hair color prediction guide developed by Walsh S. et al. [23,27]. This guide examines categorical hair color probabilities in combination with light/dark hair color shade probabilities as obtained from genotype data [23,27]. For the interpretation of skin color, we used the guide developed by L. Chaitanya et al. [16]. This guide considers the highest *p*-value in combination with the second highest *p*-value [16,28]. For example, if in the pale category we obtained a *p*-value greater than 0.7, this phenotypic characteristic depends on the effect of the second highest category if this *p*-value is greater than 0.15. In particular, it will appear darker if the second most likely category is intermediate, whereas it will appear lighter if the second most likely category is pale [16].

Prior visual phenotyping was performed by two blind assessments (two independent individuals from our laboratory not involved in this study) to verify the validity of the software prediction.

In order to verify the discrimination power of 17 human identification SNPs (not including rs9786184 and rs2032652 on the Y chromosome), we determined the random match probability (RMP) [29]. The random match probability is the estimated frequency of a genetic profile in the reference population and is calculated using allele frequencies from that population group. Therefore, the RMP is a useful value for evaluating the discriminating power of a DNA profiling system, and it indicates the probability of obtaining a match between two distinct and unrelated individuals [29].

## 3. Results

### 3.1. Genotyping of the Control Sample and Concordance

The OpenArray™ System (Thermo Fisher Scientific, Waltham, MA, USA) successfully analyzed 100% of the reference DNA samples. The average call rate and the average concordance rate were 96.9 ± 1.72 and 99.8 ± 0.56, respectively [25]. The call rate was also calculated for each SNP. The average SNP call rate was 98.9% ± 1.67, with values from 91.3–100% (Table 1).

### 3.2. Sensitivity Study

The results of the sensitivity study are shown in Table 3. A call rate higher than or equal to 90% was obtained for all samples. The results obtained demonstrated that the system is able to correctly type DNA evidence in quantities starting from 0.5 ng.

### 3.3. Genotyping of Biological Evidence from Real Cases

A total of 21 single biological evidence samples from different tissues (10 from saliva, 2 from semen, 7 from blood, 2 from urine), previously assigned to their reference perpetrator, were analyzed using the OpenArray™ System. This evidence was attributed to eight different subjects [23,24] (Table 2). A total of 17 samples presented call rates higher than 90%, with only 4 samples showing call rates below 90% (Table 2). As expected, these 4 samples were characterized by an extremely low starting concentration (Table 2).

We typed two replicates of each sample to verify the genotype call reliability. We obtained concordant results between the two replicates for all samples.

#### 3.3.1. Predicting Human Appearance from Forensic Samples

Once the correct allelic call was ascertained for all subjects, it was possible to carry out predictive analysis on the HIrisPlex-S System software [19].

The data reported in Supplementary Table S1 describe the outputs (*p*-values) obtained by the HIrisPlex-S System Software for each subject [19].

Table 2 reports the phenotype predictions given by the HIrisPlex-S System software [19].

#### 3.3.2. RMP Calculation

Calculation of the random match probability (RMP) was performed. The calculation considered 17 SNPs located on the autosomal chromosomes. The frequencies relating to the European (EUR), African (AFR), and East Asian (EAS) populations of the 1000 Genomes Project, available on the Ensembl genome browser, were used [22].

Table 4 shows the RMP values obtained for each subject.

These values show that the discriminatory power of the analyzed SNPs is not sufficient to uniquely identify a subject in the world population but can be used to verify genetic compatibility with suspects to be confirmed by traditional STR analysis [30,31].

## 4. Discussion

To the best of our knowledge this is the first application of the OpenArray™ System in the forensic field. We designed a panel of 60 SNPs for phenotype estimation and personal identification [2]. SNPs for eye, hair, and skin color prediction were directly selected from the HIrisPlex-S system, while SNPs for human identification were selected from already validated subsets (the Precision ID Identity SNP Panel (Thermo Fisher Scientific, Waltham, MA, USA) and ForenSeq DNA Signature Prep Kit (Verogen Inc, San Diego, CA, USA) [16,17,18,19]. These SNPs were first tested on 314 reference samples, revealing an average call rate of 96.9% and a concordance rate near 99.8%.

Sensitivity testing performed on serial dilutions demonstrated that samples with 0.5 ng of total input DNA can be correctly typed.

Of 21 biological evidence samples from different tissues (10 from saliva, 2 from semen, 7 from blood, 2 from urine), a total of 17 were correctly typed using OpenArray™, revealing a call rate higher than 90%.

Regarding identification purposes, since most SNPs are biallelic, they are less informative for identity testing than STR analyses [32]. Thus, many more SNPs will be needed to achieve the same level of discrimination afforded by the commonly used STR loci [33]. However, our system is not intended to replace the present STR analysis for human identification. Despite the reduced discrimination power of this system compared to STR based kits, the OpenArray™ System can be used to exclude suspects, to prioritize samples for downstream analyses providing well-established information about the prediction of eye color, hair color, and skin pigmentation.

It should be used primarily as a tool for phenotype prediction, while identification should also be supported by traditional methods. A total of 17 SNPs for human identification were used in this study, yielding a mean RMP of approximately 10^−8^.

It is important to outline that our work was intended to provide a new tool, based on previously validated SNPs, for phenotype prediction and prioritizing samples for downstream analyses for human identification [16,17,18,19,34,35]. The HIrisPlex-S panel and the related software were previously validated elsewhere [16,19]. Thus, our work was aimed at verifying the ability of OpenArray™ to correctly type the selected SNPs.

The main strengths of the OpenArray™ System are the speed of the analysis (less than four hours against the current 24–48 h), the standardized interpretation of the results, the low cost for the analysis of a single sample (about 10 euros, in the region of 20% of the current average) and the opportunity to provide both a first evaluation of biological compatibility and phenotypic information. Moreover, TaqMan chemistry is a well-known technology routinely used in the workflow of forensic laboratories to quantify the DNA extracted from biological samples.

Current weaknesses are that it is necessary to analyze the samples with a second system based on traditional STRs to determine biological compatibility. Furthermore, the OpenArray™ System is not used in the workflow of forensic genetics laboratories, limiting its availability for routine use.

## 5. Conclusions

To the best of our knowledge, this study represents the first application of the OpenArray™ System as a genotyping tool in forensic genetics. In particular, the results obtained suggest that the OpenArray™ System is robust and reliable and is suitable for use in forensic genotyping. Herein, we developed a panel for phenotypic prediction and human identification. Although the 19 SNPs used provide useful information for personal identification, a greater number of SNPs is required for the reliable identification of a subject.

The quality of the results obtained from biological evidence also promotes the application of this system in forensic laboratories to assist investigators in resolving cases.

Finally: these data suggest the applicability of this system as a genotyping method in the forensic field. In particular, it could be suitable as a first line method to verify the presence of genetic compatibility between evidence and suspects and to achieve rapid investigative information also providing well-founded predictions of eye color, hair color, and skin pigmentation. More studies will be needed for further validation of this technology and to consider opportunities to implement this custom array with more SNPs to lower the RMP and to include markers for studies of ancestry and lineage.

## Figures and Tables

**Table 1:** Single-nucleotide polymorphism (SNP) details. FDP: forensic DNA phenotyping; ID: SNPs for forensic identification [16,17,18,19].

Reference SNP Number	Assay Name	Call Rate (%)	Concordance Rate (%)	FDP/ID
rs3114908	C__27458355	100%	100%	skin
rs1800414	C___8866240_10	100%	100%	skin
rs10756819	C_____69643_10	97.8%	97.5%	skin
rs2238289	C__26683035_10	97.8%	97.5%	skin
rs17128291	C__34508041_10	100%	100%	skin
rs6497292	C__31479145_10	97.8%	100%	skin
rs1129038	C____489033_10	97.8%	100%	skin
rs1667394	C__26683195_10	97.8%	100%	skin
rs1126809	C_175675702_10	100%	100%	skin
rs1470608	C___8866144_10	97.8%	100%	skin
rs1426654	C___2908190_10	100%	100%	skin
rs6119471	C__30035872_10	100%	100%	skin
rs1545397	C__11751858_10	100%	100%	skin
rs6059655	C_189135912_10	100%	100%	skin
rs12441727	C___1289851_10	97.8%	100%	skin
rs8051733	C__43670381_20	100%	100%	skin
rs3212355	C__32390816_10	100%	100%	hair
rs312262906/rs796296176	C_339644346_10	100%	100%	hair
rs11547464	C__27541634_10	100%	100%	hair
rs885479	C___7519060_20	100%	100%	hair
rs1805008 (rs11538871)	C_175977481_10	100%	100%	hair
rs1805005	C___7519054_10	100%	100%	hair
rs1805006	C___7519055_20	100%	100%	hair
rs1805007	C___2033404_30	97.8%	100%	hair
rs1805009	C___1010501_30	100%	100%	hair
rs201326893	C_190249836_20	100%	100%	hair
rs2228479	C___2033405_20	97.8%	100%	hair
rs1110400	ANKCDY2	97.8%	100%	hair
rs28777	C___2390574_10	100%	100%	hair
rs12821256	C__26988870_10	100%	100%	hair
rs4959270	C__29334795_10	97.8%	100%	hair
rs1042602	C___8362862_10	95.7%	100%	hair
rs2402130	C__27204528_20	100%	100%	hair
rs2378249	C__16003674_10	100%	100%	hair
rs683	C___3119206_10	97.8%	100%	hair
rs16891982	C___2842665_10	100%	100%	eye
rs12203592	C__31918199_10	100%	100%	eye
rs1800407	C___8866200_10	97.8%	97.5%	eye
rs12913832	C__30724404_10	97.8%	100%	eye
rs12896399	C___3244615_10	100%	100%	eye
rs1393350	C___9491300_10	100%	100%	eye
rs10488710	C___2450075_30	97.8%	100%	ID
rs2920816	C__16156638_10	97.8%	100%	ID
rs6955448	C__29220288_10	100%	100%	ID
rs1058083	C___1619935_1_	95.7%	97.4%	ID
rs221956	C____824925_10	100%	100%	ID
rs13182883	C___2556113_10	100%	100%	ID
rs1821380	C__11673733_10	100%	100%	ID
rs6811238	C__11245682_10	100%	100%	ID
rs430046	C___9603287_10	100%	100%	ID
rs576261	C____788229_10	97.8%	100%	ID
rs10092491	C___2049946_10	95.7%	100%	ID
rs560681	C___1006721_1_	100%	100%	ID
rs1736442	C___3285337_1_	91.3%	97.3%	ID
rs4364205	C__26449463_10	95.7%	100%	ID
rs445251	C___2997607_10	100%	100%	ID
rs7041158	C__29060279_10	97.8%	100%	ID
rs13218440	C___9371416_10	100%	100%	ID
rs9786184	C__29554891_10	100%	100%	ID
rs2032652	C___2259382_10	100%	100%	ID

**Table 2:** Details of forensic biological samples. Subject A (SubjA), Subject B (SubjB), Subject C (SubjC), Subject D (SubjD), Subject E (SubjE), Subject F (SubjF), Subject G (SubjG), and Subject H (SubjH).

Sample	Source	Subject	[ng/μL]	Call Rate (%)	Predicted Phenotype	Real Phenotype
20–352	Saliva	SubjA	9.483	97.5	blue eyes; brown hair/dark hair; pale skin	confirmed
20–367	Blood	SubjA	1.263	91.7		
20–368	Blood	SubjB	2.322	93.3	blue eyes; blond hair/light hair; intermediate skin	confirmed
20–369	Blood	SubjC	6.609	97.5	brown eyes; brown hair/dark hair; dark skin	confirmed
20–361	Saliva	SubjD	18.493	95.8	brown eyes; brown hair/dark hair; intermediate skin	confirmed
20-370	Blood	SubjD	2.178	94.2		
20–364	Saliva	SubjE	36.327	96.7	brown eyes; red hair; intermediate skin	confirmed
20–371	Blood	SubjE	1.657	95.0		
20–144	Blood	SubjF	0.078	16.7	blue eyes; blond hair/light hair; intermediate skin	confirmed
20–145	Semen	SubjF	7.022	92.5		
20–146	Urine	SubjF	0.009	4.2		
20–147	Saliva	SubjF	5.534	95.0		
20–148	Blood	SubjG	4.994	96.7	brown eyes; brown hair/light hair; intermediate skin	confirmed
20–149	Semen	SubjG	0.642	52.5		
20–150	Urine	SubjG	0.012	0.8		
20–151	Saliva	SubjG	20.448	99.2		
20–065	Saliva	SubjH	18.562	97.5	brown eyes; brown hair/dark hair; intermediate skin	confirmed
20–069	Saliva	SubjH	19.640	97.5		
20–071	Saliva	SubjH	13.438	98.3		
20–072	Saliva	SubjH	12.512	97.5		
20–074	Saliva	SubjH	10.603	97.5		

**Table 3:** The call rate and concordance rate obtained from the sensitivity study.

Sample	Call Rate (%)	Concordance Rate (%)
SAMPLE_1_10 ng	100	100
SAMPLE_1_5 ng	100	100
SAMPLE_1_1 ng	100	100
SAMPLE_1_0.5 ng	100	100
SAMPLE_2_10 ng	95	100
SAMPLE_2_5 ng	98.3	100
SAMPLE_2_1 ng	96.7	100
SAMPLE_2_0.5 ng	96.7	100
SAMPLE_3_10 ng	96.7	100
SAMPLE_3_5 ng	95	100
SAMPLE_3_1 ng	90	98.21
SAMPLE_3_0.5 ng	91.7	100
SAMPLE_4_10 ng	96.7	100
SAMPLE_4_5 ng	95	100
SAMPLE_4_1 ng	91.7	98.28
SAMPLE_4_0.5 ng	95	100

**Table 4:** The random match probability (RMP) values relating to the eight subjects. European (EUR), African (AFR), and East Asian (EAS).

Subject	RMP_EUR_	RMP_AFR_	RMP_EAS_
SubjA	2.67 × 10^−8^	1.48 × 10^−8^	1.56 × 10^−8^
SubjB	5.60 × 10^−9^	1.57 × 10^−9^	3.51 × 10^−9^
SubjC	1.15 × 10^−7^	2.49 × 10^−8^	4.51 × 10_−8_
SubjD	4.24 × 10_−7_	1.91 × 10^−7^	1.37 × 10^−7^
SubjE	2.89 × 10_−8_	1.51 × 10^−8^	2.81 × 10_−8_
SubjF	1.80 × 10^−9^	6.07 × 10^−10^	3.27 × 10^−10^
SubjG	2.23 × 10_−7_	8.95 × 10^−8^	1.19 × 10^−7^
SubjH	4.26 × 10^−8^	2.30 × 10^−8^	6.44 × 10^−8^

## Data Availability

The data generated in the present study are included within the manuscript and its supplementary file.

## Supplementary Materials

The following are available online at https://www.mdpi.com/2073-4425/12/2/221/s1. Table S1. The output obtained by the HIrisPlex-S System Software for Subject A (SubjA), Subject B (SubjB), Subject C (SubjC), Subject D (SubjD), Subject E (SubjE), Subject F (SubjF), Subject G (SubjG), and Subject H (SubjH).
