# Supplementary material for: Evaluation of OpenArray™ as a Genotyping Method for Forensic DNA Phenotyping and Human Identification
Source: Genes (Basel). 2021 Feb 3;12(2):221. doi: 10.3390/genes12020221 (PMC7913479; doi:10.3390/genes12020221)
Supplement: Supplementary file 1 [file genes-12-00221-s001.pdf]

Table S1. The output obtained by the HIrisPlex-S System Software for Subject A (SubjA), Subject B (SubjB), Subject C (SubjC), Subject D (SubjD), Subject E (SubjE), Subject F (SubjF), Subject G (SubjG), and Subject H (SubjH).

	Predicted phenotypes							
	**SubjA**	**SubjB**	**SubjC**	**SubjD**	**SubjE**	**SubjF**	**SubjG**	**SubjH**
	p-value	*p-value*	*p-value*	*p-value*	*p-value*	*p-value*	*p-value*	*p-value*
**blue eye**	0.629	0.848	0	0.003	0.099	0.884	0.184	0.106
**intermediate eye**	0.288	0.088	0.007	0.054	0.134	0.073	0.15	0.153
**brown eye**	0.083	0.065	0.993	0.943	0.767	0.044	0.665	0.741
**blond hair**	0.011	0.583	0.047	0.183	0.112	0.517	0.333	0.243
**brown hair**	0.689	0.381	0.784	0.587	0.11	0.443	0.583	0.598
**red hair**	0.001	0.006	0.016	0	0.776	0.006	0.006	0.001
**black hair**	0.3	0.03	0.153	0.23	0.002	0.033	0.078	0.158
**light hair**	0.033	0.954	0.279	0.404	0.992	0.937	0.842	0.461
**dark hair**	0.967	0.046	0.721	0.596	0.008	0.063	0.158	0.539
**very pale skin**	0	0.021	0.024	0.037	0.334	0.08	0.017	0.163
**pale skin**	0.715	0.125	0.007	0.127	0.123	0.174	0.06	0.128
**intermediate skin**	0.285	0.846	0.227	0.826	0.457	0.723	0.802	0.528
**dark skin**	0	0.008	0.739	0.001	0.087	0.02	0.097	0.156
**dark to black skin**	0	0.001	0.004	0	0	0.002	0.025	0.025

Note: as an example, regarding the pigmentation of the eyes for subject A (SubjA), we obtained the following *p-values*: 0.629, 0.288 and 0.083. These values indicate that subject A has a probability of 63% of having blue eyes. As for the hair, this subject has a higher probability of having brown (*p-value*: 0.689) dark (*p-value*: 0.967) color. Pale skin of subject A showed the highest *p-value* (0.715), although it is not possible to exclude intermediate color (*p-value*: 0.285).
